# Good to Remember

**Published:** 1891-12

**Authors:** 


### GOOD TO REMEMBER.

The best statisticians place the total population of the world, in round numbers, at 1,200,000,000 to 1,400,000,000. Of these about 690,000,000 are under control of nominally Christian governments and 710,000,000 are under non-Christian governments. The total area of the habitable world is computed at about 52,000,000 square miles, of which 32,000,000 of square miles are subject to nominally Christian nations, and 20,000,000 subject to non-Christian nations. While it thus appears that three-fifths of the world's area is under the sway of nominally Christian rulers, we must set over against this encouraging feature the dark fact that not half the population is under Christian government, and only a small fraction of them have a saving knowledge of Christ.

Taking the total population of the world at the lower computation of about 1,200,000,000, the non-Christians comprise over two-thirds of the whole ; and the Christians of all classes, including the Romanists and the adherents of the Greek church, are less than one-third of the whole number. More definitely, according to one recent authority, the religions of the world are these :

| | | |
|---|---|---|
| Christians: | | |
| Romanists | 201,000,000 | |
| Protestants | 106,000,000 | |
| Greek church | 81,000,000 | |
| | | 388,000,000 |
| Non-Christians : | | |
| Buddhists | 340,000,000 | |
| Mohammedans | 201,000,000 | |
| Brahmins | 175,000,000 | |
| Confucians | 80,000,000 | |
| Shintus | 14,000,000 | |
| Jews | 7,000,000 | |
| | | 817,000,000 |
| Total | | 1,205,000,000 |

